# Even a Previous Light-Active Physical Activity at Work Still Reduces Late Myocardial Infarction and Stroke in Retired Adults Aged>65 Years by 32%: The PROOF Cohort Study

**DOI:** 10.3389/fpubh.2019.00051

**Published:** 2019-03-19

**Authors:** David Hupin, Jérémy Raffin, Nathalie Barth, Mathieu Berger, Martin Garet, Kevin Stampone, Sébastien Celle, Vincent Pichot, Bienvenu Bongue, Jean-Claude Barthelemy, Frédéric Roche

**Affiliations:** ^1^UJM-Saint-Etienne Autonomic Nervous System Research Laboratory, EA 4607 SNA-EPIS, Univ. Lyon, Saint-Etienne, France; ^2^Department of Clinical and Exercise Physiology, University Hospital of Saint-Etienne, Saint-Etienne, France; ^3^Loire-Haute Loire French Mutuality, SSAM, Saint-Etienne, France; ^4^Faculty of Medicine, UJM-Saint-Etienne, Univ. Lyon, Saint-Etienne, France; ^5^UJM-Saint-Etienne, Chaire Santé des Ainés, Univ. Lyon, Saint-Etienne, France; ^6^Support and Education Technic Centre of Health Examination Centres (CETAF), Saint-Etienne, France; ^7^French Federation of Voluntary Gymnastics (FFEPGV), Montreuil, France

**Keywords:** physical activity, sedentary behavior, work, prevention, health, cardiovascular event, cerebrovascular event, global physical activity questionnaire

## Abstract

**Background:** Work may contribute significantly to daily physical activity (PA) and sedentary behavior (SB). Physical inactivity and SB at work might be two major risk factors for premature morbidity. Therefore, the aim of this research was to describe self-reported past PA and SB at work and during leisure time within the PROOF cohort subjects, and to determine consequences of PA and SB on late health of these now retired workers.

**Material and Methods:** The PROOF cohort study was used to prospectively allow assessment of the predictive value of PA and SB at work and during leisure time among a healthy retired French population, with regard to cardiovascular and cerebrovascular events. PA (MET-h/week) and SB (h/d) were assessed using the Population Physical Activity Questionnaire (POPAQ) and the modified Global Physical Activity Questionnaire (GPAQ). Odds ratios (ORs with 95% CIs) for cardiovascular and cerebrovascular events were associated with each level of PA at work: light (<3 METs), moderate (3–5.9 METs), vigorous (≥6 METs) and were compared to SB at work.

**Results:** Out of the 1011 65-year-old subjects initially included, the 15-year follow-up has been currently completed for 688 (68%) subjects; 89 deaths (all-cause mortality, 9%) and 91 fatal and non-fatal cardiovascular and cerebrovascular events (9%), were reported. An active work (light, moderate, or vigorous intensity) was associated with a 21% reduced risk of cardiovascular (myocardial infarction) and cerebrovascular events (stroke) (OR = 0.79, 95% CI: 0.32–0.91, *p* < 0.02) compared to sedentary work. This relationship was already significant for light intensity work (32%; i.e., OR = 0.68, 95% CI: 0.31–0.87, *p* < 0.02).

**Conclusion:** There is strong causal evidence linking PA and SB at work with late cardiovascular and cerebrovascular disease. All in all, the risk for onset of myocardial infarction and stroke was lower among those who had a previous active work compared to those with previous sedentary work. Even previous light active work produced substantial health benefits.

**Clinical Trial Registration:** www.ClinicalTrials.gov, identifier: NCT00759304.

## Background

Studies from Morris et al. in the 1950s have stimulated a significant interest in understanding the relationship between physical activity (PA) and sedentary behavior (SB) at work (1). Since then, some researchers followed this twentieth century visionary with new cohort studies, which have shown that physical inactivity and SB at work are two major risk factors for morbidity and premature mortality (2–10). Transition to retirement is an important turning point in life of previously sedentary and inactive people. Indeed, a minor change in PA and SB might lead to significant health benefits (11). However, many aspects of these relationships are still poorly studied. We know that regular PA and a less sedentary lifestyle are an effective strategy for successful aging (12). A key question is the following: if one is active at work, will one always stay active during retirement? If yes, what is the eventual late health benefits? If not, what will this imply to one's health? The aim of this research was thus to describe self-reported PA and SB at work and during leisure time within the PROOF (Prognostic indicator of cardiovascular and cerebrovascular events; *ClinicalTrials.gov identifier: NCT00759304*) cohort subjects, then to determine their consequences on late health of retired workers.

## Materials and Methods

### An Observational Study: The PROOF Cohort

The PROOF cohort study was designed to prospectively assess the predictive value of autonomic nervous system activity level among a healthy retired French population, with regard to cardiovascular events and mortality (13). Subjects were recruited amongst the inhabitants of the city of Saint-Etienne, France and were eligible if aged 65 at the inclusion date. The study excluded people living in institutions. This entry age was selected since it coincides with the most frequent retirement age and consequently to the start of a new lifestyle allowing better quantification of lifestyle parameters. The PROOF study was approved by the University Hospital and the IRB-IEC (CCPRB Rhône-Alpes Loire). The National Committee for Information and Liberty (CNIL) gave its consent for data collection. All the subjects signed an informed consent for the study.

### Physical Activity and Sedentary Behavior Assessments

PA and SB were assessed during the cohort follow-up every 3 years since 2001 using (i) the Population Physical Activity Questionnaire (POPAQ) and in 2014 using (ii) the modified Global Physical Activity Questionnaire (GPAQ) for past PA and SB.

### POPAQ

The POPAQ explored the seven main dimensions of everyday life over a period of 7 days, with added specific consideration for sedentary behaviors and leisure time physical activities in our study (13). This questionnaire, validated against doubly labeled water technique and maximal oxygen consumption, was designed to provide a complete picture of a subject's usual PA (14, 15).

### GPAQ

The GPAQ was developed in 2002 by the World Health Organization (WHO) as part of the WHO STEPwise Approach to Chronic Disease Risk Factor Surveillance for PA observation. A self-administered format of the GPAQ was developed based on the original version of the GPAQ in French (available at www.who.int/ncds/surveillance/steps/GPAQ_Analysis_Guide_FR.pdf). The GPAQ, validated against accelerometry (16–18), consisted of 16 questions designed to estimate an individual's level of PA in 3 domains, with added specific consideration for work, leisure time and time spent in SB in our study. The modified version by authors was adapted from the initial self-administered questionnaire by adding an assessment of SB and each PA domain at different ages: 15, 30, 60, and 80, without rewording sentences of the validated administered version.

These questionnaires were sent by post and were self-administered in approximately 15 min. Subjects filled out the questionnaire by completing the time spent in each activity, quantifying the number of times the activity was done in a week or a day. Investigators (DH and MG) had thereafter to code the questionnaire using a specific written computer spreadsheet. GPAQ data were analyzed according to the GPAQ analysis guide of the WHO.

SB was quantified according to the number of hours/day spent sitting. PA was measured in Metabolic Equivalent of Task (MET)-minutes, which refers to the amount of energy (calories) expended per minute of PA (19). On the basis of the Ainsworth's compendium of PA, resting energy expenditure is assumed to be 1 MET. PA of 3–5.9 metabolic units (METs), is defined as moderate and PA ≥ 6 METs is considered as vigorous (19). MET values were applied to moderate (4 METs) and vigorous (8 METs) intensity variables in the work and recreation settings. These have been calculated using an average of the typical types of activity. Applying MET values to activity levels allowed us to calculate total PA expenditure. A combination of 4 METs PA for 15 min and a 6 METs PA for 15 min 5 days a week is equivalent to 750 MET-min/week.

### Fatal and Non-fatal Cardiovascular and Cerebrovascular Events Assessment

For each survey, participants received an invitation by post. Appointments were then made by phone. If the subjects did not contact us, they were contacted by phone. Medical history and examinations were taken during each clinical visit to the research center to determine clinical events and missing information obtained from hospital chart reviews and questionnaires sent to family practitioners (13).

All cause, cardiovascular and cerebrovascular mortality were established using the same procedure and by checking the national death registry for every missed medical examination. Death certificates were individually analyzed. Late fatal or non-fatal events continued to be monitored after the examination programs. In addition, new onset of cardiovascular (myocardial infarction) and cerebrovascular (stroke) events were checked for and updated at each clinical visit (13).

Data were collected on paper forms and an independent research group entered the data to ensure a double-blind data capture. The resulting database was analyzed by a statistician to identify outliers and inconsistencies. Discrepancies were then checked using the subject forms by a medical doctor to ensure statistical and medical coherence.

### Statistical Analysis

The population characteristics were analyzed using descriptive statistics and were reported as mean (±median) and frequencies (%). Chi-square was used for qualitative variables and Students *t*-test, or Wilcoxon test, for quantitative variables as appropriate. Intergroup analysis (15–30–60–80) was performed using Mann-Whitney test.

Odds ratios (OR with 95% confidence intervals, Cis 95%) for cardiovascular and cerebrovascular events (myocardial infarction or stroke) were associated with (i) each level of PA at work in early life: light (<3 METs), moderate (3–5.9 METs) and vigorous (≥ 6 METs) using chi-squared and linear regression. These data from GPAQ were compared to SB at work (reference group for which the OR was set at 1), with (ii) changes in PA at the age of 65 years. Subjects who increased or maintained their level of PA were compared to subjects who decreased their level of PA and vice versa according to POPAQ data, with (iii) changes of SB at the age of 65 years. Subjects who decreased or maintained their sedentary time were compared to subjects who increased their sedentary time and vice versa according to POPAQ data.

Changes in PA or SB were quantified as current reported value minus previous reported value for each subject at each scheduled follow-up survey (2, 3, and 4). Thus among subjects who self-reported a myocardial infarction or a stroke, change in PA or SB was measured before and in close proximity (i.e., within 3 years, which corresponded at 1 survey) of the reported cardiovascular and cerebrovascular events. For subjects with missing PA or SB data at the previous time point, the survey prior to that was selected to capture change, but only up to 1 survey prior. For instance, if a subject was missing survey 3, then change at survey 4 was quantified as survey 4 minus survey 2; however, change at survey 3 was still regarded as missing. Analyses were conducted with and without using the previous time point for missing cases to ensure consistency in results. Multivariate logistic regression analysis was performed to estimate adjusted 95% ORs. The explanatory variables were gender, age and CVD risk factors including hypertension, dyslipidemia, diabetes mellitus, overweight (BMI > 25) and smoking. All explanatory variables with *p* < 0.2 were included into the multivariate models, which were calculated using backward by elimination of non-significant variables.

Statistics were performed on R (R development Core Team, 2018), where *p* < 0.05 was considered statistically significant. All tests were two-sided.

## Results

**Figure 1:**
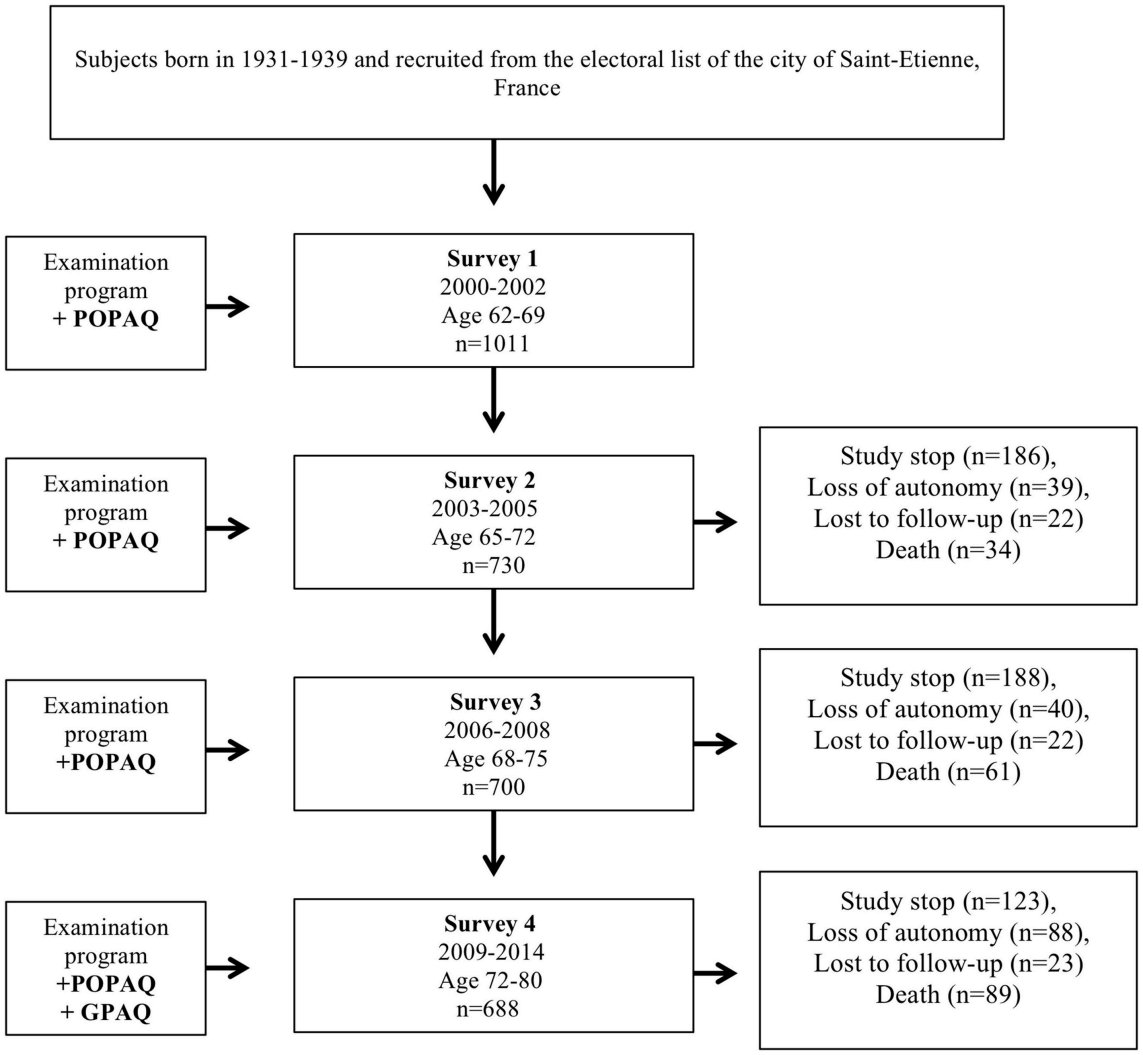
PROOF cohort study flowchart. Deaths presented as cumulative mortality from beginning of the follow-up of the cohort. Study stop represents those who did not wish to continue the study. They did not contribute information on examination program at that survey but they could return at later survey if they had changed idea. POPAQ: Population Physical Activity Questionnaire. GPAQ: Global Physical Activity Questionnaire.

Out of the 1011 65-year-old subjects initially included, the 15-year follow-up has been currently completed for 688 (68%) subjects; 89 deaths (all-cause mortality, 9%) and 91 fatal and non-fatal cardiovascular and cerebrovascular events (9%) were reported. Among the 234 remaining subjects who did not attend the last follow-up, 123 (12%) did not wish to continue the study, 88 (9%) were excluded for loss of autonomy and living in an institution (exclusion criteria), and 23 (2%) were lost to follow-up without obvious cause (Figure 1).

A majority of women (75%) were included the cohort. Sedentary work was the most represented (40% at 60 years of age), compared with light (36%), moderate (16%) and vigorous (8%) intensity work, even more than 60 years ago (36% at 15 years of age). Hypertension was one of the most common risk factors, concerning 53.5% of the subjects and 203 (29.5%) had at least three CVD risk factors (Table 1).

**Table 1:** Description of the population *(%, mean and SD)*.

**Variables**	**Age**				P^*^
	**15^⋆^**	**30**	**60**	**80^⋆^**	
	***N*** **= 688**				
Women, *n (%)*/Men, *n (%)*	514 (75%)/174 (25%)				
***N*** **(%)**					
Sedentary work	247 (36)	206 (16)	275 (40)	287 (42)	
Light intensity work	220 (32)	228 (18)	248 (36)	242 (35)	
Moderate intensity work	138 (20)	165 (24)	110 (16)	108 (16)	
Vigorous intensity work	83 (12)	89 (13)	55 (8)	51 (7)	
**Moderate-to-vigorous physical activity**, ***MEAN (± MEDIAN) MET-min/week***					
Sedentary work	2,203 (2022)	2,075 (2198)	1,833 (2004)	1,573 (1660)	–
Light intensity work	2,806 (2725)	4,410 (4352)	4,084 (3987)	3,060 (3163)	0.02
Moderate intensity work	6,367 (5321)	8,565 (7543)	5,635 (4790)	4,777 (4123)	0.009
Vigorous intensity work	7,506 (6988)	12,495 (6764)	8,722 (7123)	6,452 (6213)	0.01
**Sedentary behavior**, ***MEAN (±MEDIAN) h/d***					
Sedentary work	3.3 (3.6)	3.9 (4.4)	4.5 (3.7)	6.0 (4.7)	−
Light intensity work	3.5 (3.8)	3.8 (4.0)	3.9 (3.8)	4.3 (3.9)	0.2
Moderate intensity work	3.0 (3.0)	2.9 (3.0)	4.2 (3.9)	4.8 (4.1)	0.005
Vigorous intensity work	2.7 (2.5)	2.7 (2.9)	3.3 (3.5)	4.6 (4.0)	0.007
	−		0.03		
***P^#^***	−		0.02		
			−	0.02	

*SD, standard deviation*.

*MET, Metabolic Equivalent of Task*.

⋆ *School or work at 15 and associative activities after reaching normal retirement age*.

* *p < 0.05 between groups (light, moderate and vigorous work) and reference group (sedentary work)*.

# *p < 0.05 between (i) ≥60-year-old group and <60, (ii) 60-79-year-old group and <60, and (iii) ≥80-year-old group and 60-79*.

**Figure 2:**
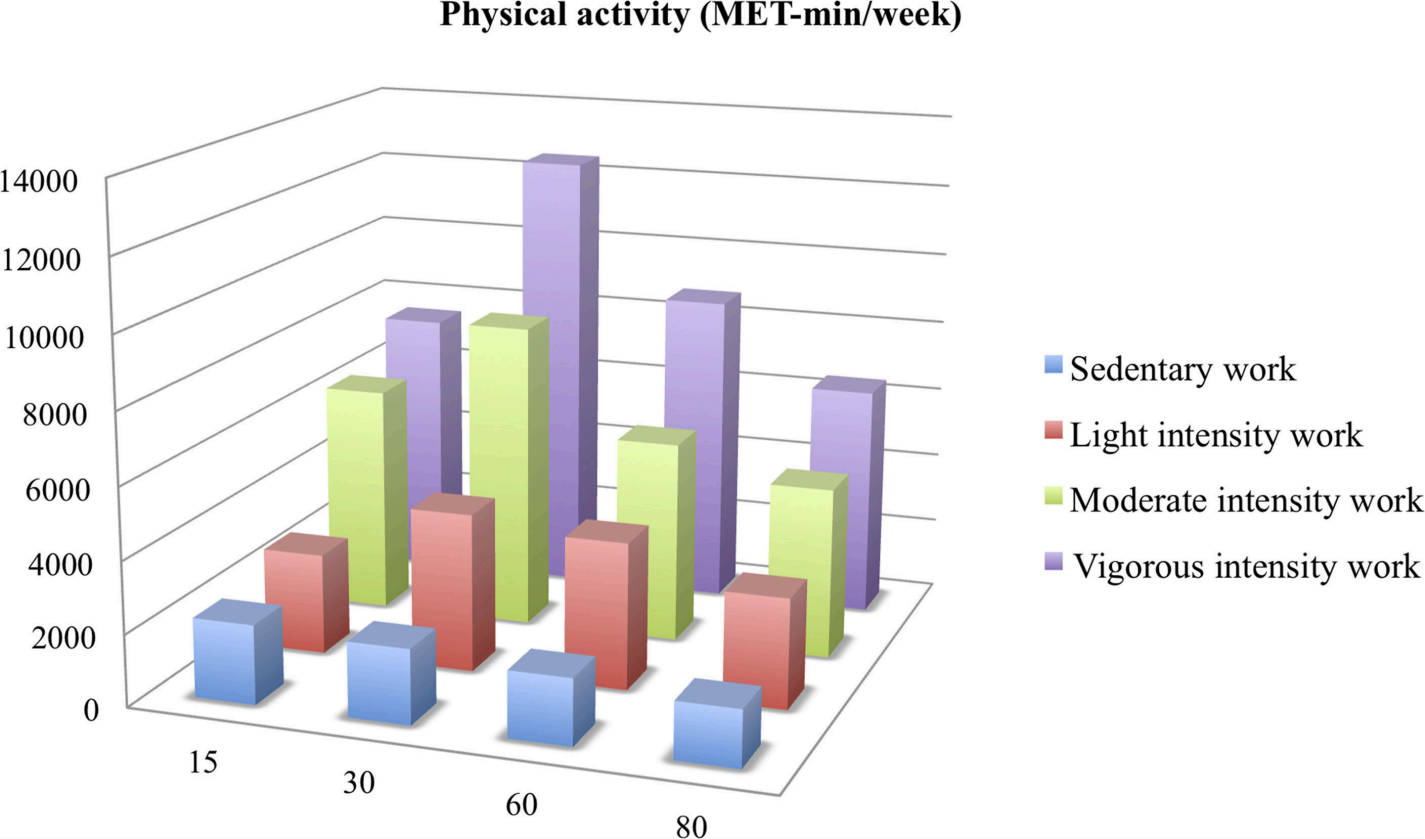
Leisure time moderate-to-vigorous physical activity (MET-min/week) from 15 to 80 years of age according to activity or not at work. MET: Metabolic Equivalent of Task.

The most active subjects at work were the most active outside the work: 8722 MET-min/week of moderate-to-vigorous physical activity (MVPA) on average at 60 years of age for subjects who had a vigorous intensity work vs. 4084 MET-min/week for a light intensity work *(p* = *0.02)*. Difference of leisure time PA between an active work (light, moderate and vigorous intensity) and a sedentary work was significant (*p* < 0.05) (Figure 2).

**Figure 3:**
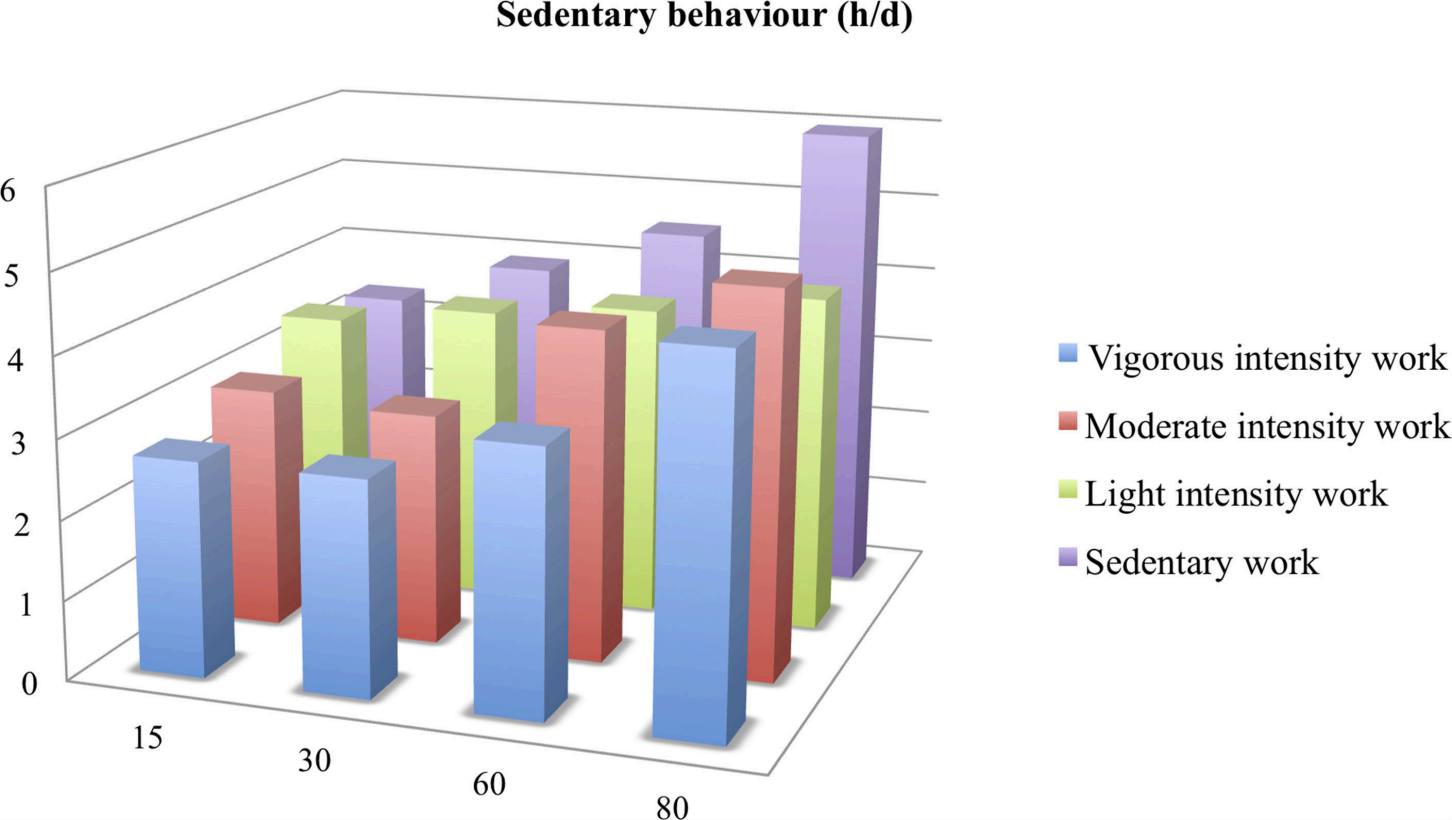
Sedentary behavior (h/d) from 15 to 80 years of age according to activity or not at work. MET: Metabolic Equivalent of Task.

Also, those who had a sedentary work remained sedentary at home: 4.5 h/d of sitting time on average at 60 years of age for sedentary workers vs. 3.3 h/d for very active workers (*p* = 0.007). In general, SBs increased with age (*p* < 0.03 between ≥ 60 and <60-year-old subjects). Even retirees who had been active at work had significant greater sedentary time from 60 years of age (*p* < 0.02 compared with <60-year-old subjects), and this was even more pronounced at age 80 (*p* < 0.02 between ≥ 80 and 60–79 year-old subjects) (Figure 3).

An active work (light, moderate or vigorous intensity) was associated with a 21% reduced risk of cardiovascular (myocardial infarction) and cerebrovascular events (stroke) (OR = 0.79 (0.32–0.91), *p* < 0.02) compared to sedentary work. This relationship was already and only significant for light intensity work (32%, i.e., OR = 0.68, 95% CI: 0.31–0.87, *p* < 0.02). Body mass index was not different between these four groups (*p* > 0.05) (Table 2).

**Table 2:** Sedentary behavior and physical activity at work: light (<3 METs), moderate (3–5.9 METs), and vigorous (≥ 6 METs) intensity and adjusted cardiovascular and cerebrovascular events odds ratios (ORs 95% CI).

**Variables**	**Total *N* at 65 = 1,011**	**Sedentary work**	**Light intensity work**	**Moderate intensity work**	**Vigorous intensity work**
*N* at 80	688	275	248	110	55
Women *n (%)*	514 (75)	209 (76.0)	199 (80.2)	76 (69.1)	30 (54.5)
BMI, *mean (±SD) kg.m^−2^*	25.0 (4.3)	25.4 (6.4)	24.9 (3.8)	24.8 (3.3)	23.8 (2.7)
BMI ≥ 25 kg.m^−2^ *n (%)*	205 (29.8)	85 (30.9)	67 (27.0)	37 (33.6)	16 (29.1)
Hypertension *n (%)*	368 (53.5)	164 (59.6)	114 (45.9)	60 (54.5)	30 (54.5)
Dyslipidemia *n (%)*	261 (38)	123 (44.7)	86 (34.7)	32(29.1)	20 (36.7)
Diabetes mellitus *n (%)*	83 (12.1)	32 (11.6)	28 (11.3)	14 (12.7)	9 (16.3)
Smoking *n (%)*	28 (4.0)	14 (5.1)	9 (3.6)	4 (3.6)	1 (1.8)
CVD risk factors ≥3, n (%)	203 (29.5)	92 (34.2)	62 (25.0)	32 (29.1)	17 (30.9)
CV events, *n (%)*	91 (9.0)	47 (17)	24 (10)	11 (10)	9 (16)
OR (95% CI) *Model A*		1	0.68 (0.31–0.87),*p* < 0.02^*^	–	–
			0.78 (0.37–0.90), *p* < 0.02^#^		
OR (95% CI) *Model B*		1	–	–	–
			0.79 (0.32–0.91), *p* < 0.02^#^		

*BMI, body mass index: weight (kg)/height (m^2^), p > 0.05 between groups*.

*SD, standard deviation*.

*Odds ratios (OR) for cardiovascular and cerebrovascular (CV) events (with 95% confidence interval) are adjusted for age and sex in Model A and for age, sex and CVD risk factors ≥3 in Model B*.

* *p < 0.05 only between light intensity work and sedentary work (reference group, OR = 1)*.

# *p < 0.05 between active group (light, moderate and vigorous work, n = 413) and sedentary work (reference group, n = 275)*.

Starting or resuming a moderate to vigorous aerobic PA during retirement was associated with a 49% reduced risk of myocardial infarction or stroke (OR = 0.51 (0.35–0.69), *p* < *0.001*). Furthermore, any reduction, even when PA was initially low, exposed older adults to an almost 2-fold increased risk of cardiovascular and cerebrovascular events (OR = 2.06 (1.47–2.88), *p* < 0.001) (Table 3).

**Table 3:** Cardiovascular end cerebrovascular events ORs (95% CI) according to changes of physical activity at the age of 65 years after 15 years of follow-up in the PROOF cohort study.

**Variable**	**Total*N* = 1,011**	**Increase or maintenance of physical activity**	**Reduction of physical activity**
Population (%)	688	354 (51%)	334 (49%)
Women *n (%)*	514 (75)	261 (73.7)	253 (75.7)
BMI, *mean (±SD) kg.m^−2^*	25.0 (4.3)	24.9 (4.9)	24.8 (4.9)
BMI ≥25 kg.m^−2^ *n (%)*	205 (29.8)	97 (27.4)	108 (32.3)
Hypertension *n (%)*	368 (53.5)	160 (45.2)	208 (62.3)
Dyslipidemia *n (%)*	261 (38)	106 (29.9)	155 (46.4)
Diabetes mellitus *n (%)*	83 (12.1)	17 (4.8)	66 (19.7)
Smoking *n (%)*	28 (4.0)	16 (4.5)	12 (3.6)
CVD risk factors≥3, *n* (%)	203 (29.5)	79 (22.3)	124 (37.1)
CV events (%)	91 (9.0)	32 (9.0)	59 (17.7)
OR (95% CI) *Model A*		1	1.89 (1.2–2.98), *p* < 0.01
		0.53 (0.34–0.83), *p* < 0.01	1
OR (95% CI) *Model B*		1	2.06 (1.47–2.88), *p* < 0.001
		0.51 (0.35–0.69), *p* < 0.001	1

*BMI, body mass index: weight (kg)/height (m^2^), p > 0.05 between groups*.

*SD, standard deviation*.

*Odds ratios (OR) for cardiovascular and cerebrovascular (CV) events (with 95% confidence interval) are adjusted for age and sex in Model A and for age, sex and CVD risk factors ≥3 in Model B*.

Reducing a SB during retirement was associated with a 30% reduced risk of myocardial infarction or stroke (OR = 0.70, 95% CI: 0.36–0.90, *p* < 0.02). Furthermore, any increase in SB, even when sedentary time was initially low, exposed older adults to an almost 1.5-fold increased risk of cardiovascular and cerebrovascular events (OR = 1.24, 95% CI: 1.11–2.82, *p* < 0.02), without difference in body mass index between both groups (*p* > 0.05) (Table 4).

**Table 4:** Cardiovascular end cerebrovascular events ORs (95% CI) according to changes of sedentary behavior at the age of 65 years after 15 years of follow-up in the PROOF cohort study.

**Variable**	**Total *N* = 1,011**	**Reduction or maintenance of sedentary behavior**	**Increase of sedentary behavior**
Population (%)	688	299 (43%)	389 (57%)
BMI, *mean (±SD) kg.m^−2^*	25.0 (4.3)	24.6 (5.1)	25.1 (4.9)
BMI ≥ 25 kg.m^−2^ *n (%)*	205 (29.8)	94 (31.4)	111 (28.5)
Hypertension *n (%)*	368 (53.5)	169 (56.5)	199 (51.1)
Dyslipidemia *n (%)*	261 (38)	126 (42.1)	135 (34.7)
Diabetes mellitus *n (%)*	83 (12.1)	34 (11.4)	49 (12.6)
Smoking *n (%)*	28 (4.0)	10 (3.3)	18 (4.6)
CVD risk factors ≥3, *n* (%)	203 (29.5)	88 (29.4)	115 (29.5)
CV events (%)	91 (9.0)	29 (9.7)	62 (15.9%)
OR (95% CI)		1	1.24 (1.11–2.82), *p < 0.02*
		0.70 (0.36–0.90), *p < 0.02*	1

*BMI, body mass index; weight (kg)/height (m^2^), p > 0.05 between groups*.

*Odds ratios (OR) for cardiovascular and cerebrovascular (CV) events (with 95% confidence interval) are adjusted for age and sex*.

## Discussion

### Principle Findings

This study found that the risk of cardiovascular and cerebrovascular events in the PROOF cohort was related to their 15–60 years sedentary or active work as well as to their SB and leisure time PA during the 15 years of follow-up of our current cohort of 80-year-old subjects. Indeed, an individual's occupational activity may contribute significantly to daily PA and SB (13), even in retired people.

In this context, the risk for the onset of myocardial infarction and stroke was lower among those who had an active work compared to those with sedentary work. Even a light active work produced substantial health benefits, which is important since retired people tend to present with more sedentary and less active behaviors.

Interestingly, being sedentary at work was associated with more SB outside of work; while on the contrary, being active at work was associated with more leisure PA outside of work. Some studies already showed that sedentary workers were more likely to have high leisure time sitting. On the contrary to our study, some other studies showed that active work was associated with more sedentary leisure time (11, 20, 21). Hence, the relationship between PA at work and SB outside work is still unclear.

Moreover, we found that sedentary workers who reported the highest sitting time at work also reported the lowest leisure time PA. Thus, sedentary work was not compensated with less SB outside of work. Worse still, there would even be an accumulating sedentary effect of a physical inactivity. This finding is supported by some other studies with a similar focus (9, 11, 12, 20–24).

The decrease in PA and increase in SB with age observed after 60 years is in accordance with some previous studies in healthy subjects and can be explained both with the decrease in daily PA (20, 24). Retirement is an important turning point in life with many changes in daily routines. These results suggest that the lack of time outside the work period to practice PA is not a valuable excuse. This is important since we demonstrate, as others, that even after retirement, the decrease in PA and increase in SB may contribute to an increased risk of cardiovascular and cerebrovascular morbidity (12, 20). The explanatory variables for cardiovascular and cerebrovascular events (myocardial infarction or stroke) were gender, age (Model A in Table 2) and CVD risk factors ≥ 3 (including hypertension, dyslipidemia, diabetes, overweight, and smoking, Model B in Table 2).

We did not find a significant link between moderate or vigorous intensity at work and cardiovascular and cerebrovascular events, which is rather inexplicable because this relationship was present for light intensity work (ORs only adjusted with age and sex, *model A*). (i)This may be explained by the limited number of cardiovascular and cerebrovascular events in the cohort. (ii) Also, isolated CVD risk factors were not significant to explain cardiovascular and cerebrovascular events probably due to an age effect on these risk factors and probably due to their low prevalence in this relatively healthy population (smoking and diabetes mellitus). More probably, (iii) the explanation may lie in the J-shaped dose–response relationship between occupational PA and all-cause mortality, which have previously been reported for moderate-to-vigorous PA. Indeed, a very recent meta-analysis of 193,696 participants showed that men engaging in high (compared to low) level occupational PA have an 18% increased risk of all-cause mortality, even after adjustment for relevant confounders, such as leisure time PA (25). However, no dose-effect was identified in our study. It would seem that cardiovascular end cerebrovascular events might be more present in subjects from the cohort with at least 3 CVD risk factors.

### Strengths and Limitations

The main strengths of our study include the PROOF cohort based design, with the long follow-up after retirement, allowing people to reach ages with a high morbidity-mortality rate, as well as the repeated measurements of PA and SB, and strong and complete information on morbidity-mortality (13).

However, there are some limitations to our study. This study relied on self-reported PA and SB (via modified GPAQ), which are subject to recall and information bias, especially for older people over the age of 80 in our study, possibly resulting in under-reporting of SB and over-reporting of PA. However, there is no reason to assume that these biases would be different for the different age periods and for the different work classes (26, 27). Also, simple questionnaires are well-suited for the study of changed behavior in large epidemiological studies (GPAQ) and the repetition of identical questions at each survey is one of the main strengths of our study (POPAQ).

The GPAQ is commonly used in Western and in low/middle income countries. According to the WHO, the GPAQ has been administered in more than 100 countries, initially developed for face-to-face interviews (www.who.int/chp/steps/GPAQ/en/index.html) (28). GPAQ was developed to combine the strengths of the short and the long International Physical Activity Questionnaire (IPAQ) but being considerably shorter (16 items) than the long IPAQ (27 items). It provided reproducible data and showed a moderate-strong positive correlation with IPAQ, the previously validated and accepted measure of PA (16, 17, 29). The French GPAQ format has been validated vs. actimetry (18, 30). There was a moderate/acceptable correlation for MVPA (spearman correlation, *r* = 0.47, from 0.45 to 0.63 in other study), often higher than reported in other studies (16, 17, 29). Correlation was low for sitting time, without difference with previous studies. The self-administered GPAQ has already studied (27, 31). Findings show that both interviewer- and self-administered modes of the GPAQ are comparable (16, 28).

However, accelerometers have limitations such that they do not measure water activities or cycling, so they may also underestimate MPVA (16, 18, 29, 31). Therefore, it may be relevant to use both a subjective and an objective measurement tool to obtain an assessment of PA type, intensity, duration, frequency and context. Measuring SB was difficult. Questionnaires explored a wide range of self-reported SBs during work and leisure. More accurate measurement might be provided by using an objective measurement tool that could distinguish between postures.

### Implications for Policy and Practice

Epidemiological studies are a powerful tool for scientific societies to promote recommendations regarding PA intended for the general population. Based on our results, we propose to promote even a low intensity of PA at work, which corresponds in practice to advising sedentary people at work to get up regularly from the chair and walk a few minutes (to go down and to go up the stairs several times a day after a long sitting time, at the break or between two meetings) would be an already relevant advice (32–34).

### Key Message

For the time being, it seems appropriate to recommend that even a low intensity active work should be encouraged as a means to reduce cardiovascular and cerebrovascular diseases. The positive correlation between physical activity at work and leisure time physical activity indicated that these two types of exercise may be a potential intervention to prevent myocardial infarction or stroke regardless of their activity intensity at work (35, 36).

We could say loud and clear that it is never too late to be physically active! It is not all over now! Our data may help to better identify target groups in public health interventions to specifically reduce SBs in retired adults. We should hearten everybody to aim at the current WHO recommendations through the whole spectrum of PA.

## Conclusion

There is strong causal evidence linking both PA and SB at work and after retirement with cardiovascular or cerebrovascular disease. Even a light active work produced late substantial health benefits. Thus, the promotion of PA aims to lead retirees with an intention to change, to accompany them in this change, and to encourage them to maintain a more active lifestyle in the long term.

## Author Contributions

DH had full access to all data in the study and takes responsibility for the integrity of the data and the accuracy of the data analysis. DH, FR, and J-CB had the idea for and designed the study. All authors had a substantial contribution to the conception and design. DH and JR conducted the literature search. DH, FR, and J-CB developed the study methodological design. DH, FR, and J-CB were responsible for collection and analysis of data. DH provided statistical expertise. DH, FR, and J-CB were responsible for interpretation of data, drafted the manuscript and submitted the paper for publication. DH, FR, JR, NB, MG, MB, KS, SC, VP, BB, and J-CB critically revised the manuscript for important intellectual content. All authors approved the final version.

### Conflict of Interest Statement

The authors declare that the research was conducted in the absence of any commercial or financial relationships that could be construed as a potential conflict of interest.
